# Improving the Prehospital Identification and Acute Care of Acute Stroke Patients: A Quality Improvement Project

**DOI:** 10.1155/2022/3456144

**Published:** 2022-02-09

**Authors:** Huan Bao, Sumian Zhang, Junjie Hao, Lian Zuo, Xiahong Xu, Yumei Yang, Hua Jiang, Gang Li

**Affiliations:** ^1^Department of Neurology, Shanghai East Hospital, Tongji University School of Medicine, Shanghai 200120, China; ^2^Department of ICU, Shanghai East Hospital, Tongji University School of Medicine, Shanghai 200120, China; ^3^Department of Medical Education, Shanghai Pudong Medical Emergency Center, Shanghai 201206, China

## Abstract

**Background:**

There are a large number of stroke patients in China, and there is currently a lack of prehospital acute stroke care training programs.

**Aim:**

To develop a prehospital emergency medical service (PEMS) training program to improve the prehospital identification and acute care of acute stroke.

**Methods:**

Forty prehospital emergency doctors whose service stations are located within a 10 km radius from Shanghai Pudong New Area Medical Emergency Service Center took this course on November 13, 2014. A questionnaire was designed to evaluate the PEMS personnel's knowledge in stroke and acute stroke care and was conducted before and after training as an assessment of the effectiveness of training. The patient population in this study included a baseline cohort before training and a prospective cohort after training, each composed of patients who were sent to Shanghai East Hospital South Stoke Center within one year. The transit time, final diagnosis, administration of thrombolysis, and door-to-needle time (DNT) were collected and analyzed.

**Results:**

After the training, 100% of the PEMS personnel were competent to identify stroke cases using the Cincinnati prehospital stroke scale (CPSS). All participants realized that intravenous thrombolysis therapy in a time-sensitive manner is the most effective way to treat acute ischemic stroke. Although there was no difference in first-aid transit time before and after training, the stroke diagnosis rate improved by 6.5% after training (*P*=0.03). The thrombolysis rate increased to 29.6% from 24.3% but did not reach statistical significance. Compared to 84.0 minutes (standard deviation: 23.1 minutes) before the training, the average DNT after training was 53 minutes (standard deviation: 15.0 minutes), demonstrating a remarkable reduction (*P* < 0.01).

**Conclusion:**

The training program effectively improved the PEMS personnel's knowledge in stroke and stroke acute care.

## 1. Introduction

The Chinese National Center for Cardiovascular Disease reported that there were 13 million stroke patients in 2018 [1]. Stroke and ischemic diseases were the leading causes of death and DALYs at the national level in China in 2017 [2]. The economic burden of stroke in China was severe [3]. While intravenous thrombolytic therapy (rt-PA) becomes the most effective treatment for acute ischemic stroke [4, 5], the time window from the onset of symptoms to deliver thrombolysis drug is critical. Studies have shown that thrombolysis within 2 hours can significantly reduce the risk of 3-month mortality by 37%, and thrombolysis within 2 to 4.5 hours can significantly reduce the risk of 3-month mortality by 26% [6]. Even the time window for recombinant tissue-type plasminogen activator (rt-PA) treatment can be extended up to 4.5 hours since stroke onset, “time is brain” principle still drives service providers to pursue the reduction of any source of delay [7]. Every 15-minute decrease in treatment delay provided an average equivalent of 1 month of additional disability-free life [8]. According to the report of the China cerebrovascular disease monitoring platform, the incidence of intravenous thrombolysis within 3.5 hours of hospitalized patients with ischemic stroke in 2018 was 24.2%, and the hospital mortality rate was 0.4% [1]. However, many studies from China mainly focused on risk factors of prehospital delays, such as demographic characteristics, region disparity, and access to medical resources [9, 10]. Few studies investigated the role of prehospital emergency medical service (PEMS) in detail.

The purpose of this study is to design a PEMS training program to improve the knowledge level of emergency personnel so as to improve the quality of prehospital first aid for stroke patients in China and promote the further development of prehospital first-aid training system. This study adhered to the Standards for QUality Improvement Reporting Excellence [11] checklist for reporting.

## 2. Methods

### 2.1. PEMS Training Program

The training materials were adapted from 2010 American Heart Association (AHA) Guidelines for Cardiopulmonary Resuscitation and Emergency Cardiovascular Care Science Part II: Adult Stroke [12], with a focus on critical care 1 hour before the hospital admission. The materials included a textbook for trainees, an instructor's book, and 25-minute videos, which covered the basic knowledge of stroke and stroke-related prehospital acute care. The training process included three sessions, with a combination of video learning, group discussion, and quiz. The discussion took place at the end of each session. Each discussion was about 3 topics from the following list: 1) the clinical presentation, signs, and symptoms of acute stroke; 2) type of stroke; 3) how to identify the type of stroke in earlystage; 4) identify acute stroke by PEMS using the CPSS; 5) why should the patient be sent to the nearest stroke center? 6) what examination should be performed during transportation? 7) why is it critical to communicate with the emergency department in the hospital earlier? 8) how to contact with stroke center? 9) what should be reported in the stroke center, such as intravenous thrombolytic time window, indications, and contraindications.

### 2.2. Implementation and Evaluation

Participants of the training program were open to all emergency doctors from service stations within a 10 km radius from Shanghai Pudong new area medical emergency service center, including 8 suburban stations: Zhoujiadu station, Yangsi station, Zhoupu station, Punan station, Northcai station, Sanlin station, Cambridge substation, and Renji substation. Five emergency doctors from each station, including the captain, eventually joined the training program. All these 40 emergency doctors have a medical degree, board certificate, and 1 to 8-year experience in PEMS. The instructors are vascular neurologists from Shanghai east hospital south stroke center, who are certified AHA ACLS course directors. The course was given in December 2014. Attendance was mandatory to ensure 100% compliance with training sessions.

A questionnaire was designed to test the participants' knowledge using 15 questions (supplementary table S1) before and after training.

### 2.3. Data Collection

This study included a baseline period before training, which was from December 1^st^, 2013 to November 30^th^, 2014, and a posttraining period from December 1^st^, 2014 to November 30^th^, 2015. Patients included all suspected stroke patients who were sent to a stroke center during the study. First-aid transit time was defined as the time elapsed between the dispatcher receiving a call from the EMS system and the ambulance arriving at the hospital and was recorded. The clinical diagnosis information was also collected, such as thrombolysis, DNT time, and final diagnosis. The diagnosis of stroke was determined by a neurologist based on head CT or MRI results. Whether there are differences in the first-aid transit time, diagnosis rate, thrombolysis rate, and DNT time between the two groups of patients were analyzed.

### 2.4. Statistical Analysis

SPSS.21.0 statistical software was used for data analysis, using two-sided, *P* < 0.05 as statistical significance cut off. Continuous variables were summarized as mean ± standard deviation. Categorical data were as frequency and percentage. Student's *t*-test or Chi-square test was used for precomparison and postcomparison, as appropriate.

## 3. Results

### 3.1. The Improvement of Stroke-Related Emergency Care Knowledge after Training

Before the training program, the PEMS physicians showed inadequate knowledge in acute stroke and the related emergency care. Among them, 26.7% thought that prehospital diagnosis of stroke was not easy; 73.3% determined stroke event based on experience. In terms of the choice of hospital, 86.7% of the participants would be impacted by families' preference. After training, 100% of the participants were capable of using CPSS to evaluate the suspected stroke, while 97.7% agreed that the patients with acute stroke should be sent to the nearest stroke center. Finally, after training all the participants reached a consensus that the time window for intravenous thrombolysis is critical to treat acute ischemic stroke.

### 3.2. Changes in First-Aid Transit Time

Table 1 compares the key components of first-aid transit time before and after training. A total of 409 suspected stroke patients were sent to the stroke center during the baseline period, comparing to 446 suspected patients during the posttraining period. The total transit times did not differ before and after training (mean and SD: 29.58 ± 10.00 minutes vs. 29.29 ± 8.87, respectively, *P*=0.65). Although there was no significant difference in each time component as well, of note, the average transport time was shortened by about half a minute after training (*P*=0.11).

### 3.3. Quality Improvement of Emergency Treatment for Emergency Patients

Among 409 suspected stroke patients sent to the stroke center, 100 (24.4%) were diagnosed as a stroke. The accuracy increased significantly to 138 diagnosed cases out of 446 suspected patients (30.9%), *P*=0.03 (Table 2). There was no significant difference in the proportion of hemorrhagic stroke and ischemic stroke during periods before and after training (*P*=0.54). The success rate of intravenous thrombolysis for ischemic stroke did not change significantly during and after training period (18/74 or 24.3% vs. 32/108 or 29.6%, before and after, respectively, *P*=0.43). However, the DNT time for hemorrhagic stroke was significantly improved by half-hour shorter (84.00 ± 23.10 minutes vs. 53.03 ± 15.01 minutes, before and after, respectively, *P* < 0.001) (Table 2).

## 4. Discussion

In this study, we designed a training course to improve the PEMS personnel's knowledge and skill in the early identification of suspicious stroke cases. The results show that the PEMS training program effectively improved the PEMS personnel's knowledge and skill in prehospital acute care for acute stroke patients. The diagnosis accuracy rate of acute stroke improved by 6.5% after training (*P*=0.03). The average DNT time was 30 minutes shorter than that of before (*P* < 0.001).

The incidence of intravenous thrombolysis within 3.5 hours in hospitalized patients with ischemic stroke in China in 2018 was 24.2% [1]. The prehospital delay and the lack of prompt response in emergency department were the major factors contributing to this low rate [14]. Jiang et al. reported in 2016 that about 1/3 of the patients with stroke were failed to be identified on ambulance [9]. In contrast, starting from 2008, stroke fell to the fourth leading cause of death from the third place in the United States, as a result of improvement in the earlier treatment of acute stroke. As the first-aid resource for most stroke patients, the emergency medical services (EMS) has been playing a vital role in stroke identification and the further delivery of thrombolytic therapy [15]. Many studies have shown that rapid EMS response and prehospital notification can significantly increase thrombolysis rates and shorten the DNT time [16–19]. In addition, many countries have initiated national stroke registries for the sake of improving the quality of stroke care [20, 21]. In line with the registries, there were many initiatives aiming at providing better acute management of stroke have been put in place in the developed countries. However, there is no training program for prehospital acute stroke care in China. Earlier identification of stroke syndromes and delivery of prehospital care remain big challenges. In this study, we adapted the contents related to ischemic stroke from the American Society of Advanced Life Support course to train the first-aid staff in the Shanghai Pudong New Area, aiming to improve the quality of prehospital care for patients with acute stroke.

There are many prehospital screening scales for stroke. The CPSS is a standardized and easy-to-use stroke screening tool. It is recommended to implement in emergency systems for prehospital assessment of stroke [22, 23]. The meta-analysis by De et al. [22] showed that the diagnostic sensitivity and specificity of CPSS were 82.46% and 56.95%, respectively. They also found that there were no significant differences in terms of sensitivity when CPSS was performed by physicians or nonphysicians. In our study, the diagnosis accuracy of PEMS personnel has significant improvement after training (from 24.4% to 30.9%, *P*=0.03). Despite all this, the accuracy is still at a very low level. The American Heart Association (AHA)/the American Stroke Association (ASA) Guidelines provide four validated and standardized tools for prehospital stroke screening: CPSS, Recognition of Stroke in the Emergency Room (ROSIER), Los Angeles Prehospital Stroke Screen (LAPSS), and FAST, but no recommendation was given about which is better to use [5]. In the latest comparative study, Alijanpour et al. [24] compared the sensitivity and specificity of six scales: FAST, CPSS, LAPSS, Los Angeles Motor Scale (LAMS), and The Finnish Pre-hospital Stroke Scale (FPSS), and failed to find which scale is better for early identification of stroke. So did Saberian [25]. Therefore, the available evidence does not prove which tool is more suitable yet. The gold standard for prehospital acute stroke screening still needs more researchers to explore.

Our results showed that the training program significantly improved stroke care-related knowledge. For example, they all mastered the CPSS as a tool for early evaluation and agreed on the critical importance of the time window for intravenous thrombolysis. As a result, the diagnosis accuracy rate of acute stroke increased significantly by 27% relatively (*P*=0.03). The average DNT time was 30 minutes shorter than that of before (*P* < 0.001). This improvement was also a benefit of the early alert sent to the stroke center from PEMS staff. Although we were able to achieve a mean DNT of 53 minutes, however, it was still far worse than what has been achieved in the developed countries. A Fenland study reported the achievement of 20 minutes DNT in 2011 [26]. The integration of the existing “code stroke model” and the Helsinki stroke thrombolysis model resulted in a DNT of 25 minutes in Melbourne [27]. A USA study reported ≤39 minutes DNT after developing a streamlined intravenous tPA protocol following a lean process improvement methodology [28].

There were no statistically significant changes in duration and each time component after training. In order to ensure the accuracy of data collection, all the timers or clocks used for recording time were synchronized. In this study, the average dispatch times were similar before and posttraining (2.58 minutes vs. 2.53 minutes on average, respectively, *P*=0.75). The average response time before and after training was 9.55 minutes vs. 9.46 minutes (*P*=0.77). The average scene time before and after training was 8.49 minutes vs. 8.31 minutes (*P*=0.52). The average transport time was 8.96 minutes vs. 8.31 minutes (*P*=0.11). All those time components complied with our protocol. Of note, the scene time was about half of 15 minutes, which is the required time according to protocol.

There were some limitations to this study. We did not analyze the distance from the ambulance departure to the scene and from the scene to the stroke center. Therefore, we did not know the impact of the distance to transit time. Although the thrombolysis rate changed from 24.3% to 29.6%, representing a 5% absolute increase of 20% relative increase, it did not reach statistical significance. Therefore, a long-term survey is needed to accumulate a larger sample size and reassess this change. Finally, the accuracy of stroke diagnosis needs to be improved. In the future, we will include more sample sizes and explore the best prehospital stroke screening scale that can improve the diagnostic accuracy of PEMS personnel.

## 5. Conclusion

The PEMS training program effectively improved the PEMS personnel's knowledge and skill in prehospital acute care for emergency stroke patients. The accuracy rate of stroke diagnosis increased significantly after training, and the average DNT time significantly shortened. The results suggest that after training, the quality of acute care for stroke was improved.

## Figures and Tables

**Table 1:** Comparison of first-aid transit time before and after training.

Time components ^*∗*^	Before training *N* = 409	After training *N* = 446	*P-*value
Dispatch time	2.58 ± 2.43	2.53 ± 2.29	0.75
Response time	9.55 ± 4.72	9.46 ± 4.49	0.77
Scene time	8.49 ± 4.39	8.31 ± 3.87	0.52
Transport time	8.96 ± 6.76	8.31 ± 5.12	0.11
Total transit time	29.58 ± 10	29.29 ± 8.87	0.65

^*∗*^The definition of time components [13]: Dispatch time: the time elapsed from receiving a call for emergency service to dispatch a PEMS crew. Response time: the time elapsed from ambulance departure to arrival to the site of an ill person. Scene time: the time elapsed from the arrival of the PEMS crew on the scene until departure. Transport time: the time elapsed from departure to arrival at a hospital. Total transit time: the sum of the above four components.

**Table 2:** Comparison of stroke-related clinical parameters before and after training.

Parameters	Before training	After training	*P*-value
Diagnose accuracy	24.4% (100/409)	30.9% (138/446)	0.03
Type of stroke			0.54
Hemorrhagic stroke	26 (26%)	30 (21.7%)	
Ischemic stroke	74 (74%)	108 (78.3%)	
Thrombolysis rate	24.3% (18/74)	29.6% (32/108)	0.43
DNT time, min	84.00 ± 23.10	53.03 ± 15.01	<0.0001

## Data Availability

The datasets used and/or analyzed during the current study are available from the corresponding author on reasonable request.

## Supplementary Materials

Supplementary Materials Table 1. Questionnaire.
