# Supplementary material for: Improving the Prehospital Identification and Acute Care of Acute Stroke Patients: A Quality Improvement Project
Source: Emerg Med Int. 2022 Feb 9;2022:3456144. doi: 10.1155/2022/3456144 (PMC8850070; doi:10.1155/2022/3456144)
Supplement: Supplementary Materials — Table 1. Questionnaire. [file 3456144.f1.docx]

**Supplementary Table 1. Questionnaire**

| 1. Do you think it is easy to diagnose stroke before hospital? Y/N  2. Do you adjudicate stoke event by experience? Y/N  3. Are you able to use CPSS to evaluate the suspected stroke? Y/N  4. Which of the following factors will impact your choice of emergency care hospital? A) Opinion from patient’s family members B) Distance to the hospital  5. How do you handle the inference from patient’s family members regarding to the choice of emergency care hospital? A) Follow their choice B) Suggest the nearest stoke emergency center  6. What kind of hospital will you send patient to? A) A hospital providing thrombolytic treatment B) Any nearest hospital  7. What do you think is the most effective treatment for acute ischemic stroke?  A) Intravenous thrombolysis within time window B) Conventional medication  8. Which is an intravenous thrombolytic drug that you know to treat acute ischemic stroke? A) Alteplase (rt-PA) B) Urokinase C) Other  9. What types of stroke do you know? A) Hemorrhagic stroke B) Ischemic stroke C)Both  10. Do you know how to determine the type of stroke? Y/N  11. As an EMS personnel, what are the most critical first aid measure for patients with acute stroke? (Open text)  12. Do you know the time window for thrombolytic therapy? Y/N  13. Do you know the indications for intravenous thrombolytic therapy? Y/N  14. Do you think if it is necessary to notify the emergency department before you send patient to hospital? Y/N  15. Can you accurately determine the time when patient's symptoms started? Y/N |
| --- |
